# In Reply to: Queries Regarding Local Erythropoietin Injection in Tibiofibular Fracture Healing

**DOI:** 10.5812/traumamon.14208

**Published:** 2013-09-10

**Authors:** Ali Nemati, Amir Hossein Fallahi

**Affiliations:** 1 Department of Orthopedics, Emam Hosein Medical Center, Shahid Beheshti University of Medical Sciences, Tehran, IR Iran

**Keywords:** Fractures, Bone, Erythropoietin, Wound Healing

*Dear Editor,*

*In reply*- We would like to thank Dr. Duedal Rölfing for his keen observations. We hope that this study encourages prospective scientific publications. Erythropoietin (EPO) is a glycoprotein produced by the kidney which promotes the formation of red blood cells in the bone marrow. Recent studies have determined additional roles of EPO in tissue survival and cellular proliferation (1-3). EPO may accelerate bone formation and contribute to fracture healing (2). Despite this fact, the subject is still in debate (3). Tibial fractures are of the most common fractures in orthopedics and delayed union in long bones is mostly seen in the Tibia (4, 5). Not only is EPO effective in bone healing, but also it is effective in the long term (2). We have done a five-year long-term study and EPO dose was chosen based on our experience in a pilot study. Our treatment regime was as EPO (4000 IU), three ampoules were injected in one group into the fracture site two weeks after the operation and under sterile conditions and guide of C-arm. However, in obese patients (BMI > 30) we injected five ampoules. Of course, we consulted with the EPO manufacturer and used their recommendations. Based on the pilot study which lasted one year, we concluded lower dosages in obese patients were not sufficient and we used higher dosages in our recent study. Patients with multiple lower injuries, metaphyseal and isolated tibial, pathologic, comminuted, osteoporotic and open fractures were excluded. Also patients under treatment with steroids, anticoagulants, nonsteroidal antiinflammatory drugs, calcium channel blockers and nicotine were also excluded from the study. All patients underwent surgical ﬁxation by close intramedullary nailing. All of our cases were patients with closed fracture type A,B of AO classification including all subgroups of A1,A2,A3 and B1,B2,B3.There was no significant difference between case and control groups in soft tissue healing. The patients were followed after EPO injection by physical examination and radiographic imaging every four weeks until union was conﬁrmed. According to the radiographic studies, the mean union period was 19.35 ± 2.66 weeks in the EPO group compared to 21.50 ± 3.18 in the placebo group (P = 0.01). As the mean time was 20 weeks for union, we did five x-ray exam per patient with four weeks intervals which has been recommended (6).
